# When the Right and Left Coronary Arteries Join: A Case Report of Intercoronary Communication and Literature Review

**DOI:** 10.1155/cric/8331602

**Published:** 2026-10-06

**Authors:** Yassine Ettagmouti, Amine Bejar, Asmaa Mounssif, Ayoub Huda, Nabil Poulos, Pierre Aubry

**Affiliations:** ^1^ Department of Cardiology Centre Hospitalier de Gonesse Gonesse France; ^2^ Department of Cardiology International Clinic Casablanca Morocco; ^3^ Department of Cardiology Hôpital Bichat Assistance Publique-Hôpitaux de Paris Paris France aphp.fr

**Keywords:** anomalous coronary artery, case report, coronary angiography, intercoronary communication

## Abstract

An intercoronary communication is a very rare congenital anomaly characterized by a short connection between two main coronary arteries. A 41‐year‐old female presented with dyspnea and elevated D‐dimer and N‐terminal pro brain natriuretic peptide levels. Pulmonary CT angiography demonstrated a pulmonary embolism and significantly enlarged mediastinal lymph nodes. Echocardiography revealed apical akinesia and impaired left ventricular function (ejection fraction of 45%). Coronary angiography showed no evidence of obstructive coronary artery disease but identified an abnormal continuity between the distal right coronary artery and the distal left circumflex artery. Each coronary injection demonstrated retrograde filling of the contralateral artery. The short intercoronary connection did not have the characteristics of an acquired collateral circulation. Cardiac MRI revealed apical akinesia with multifocal areas of subepicardial late gadolinium enhancement raising suspicion for cardiac sarcoidosis. Fewer than 30 cases of intercoronary communication have been reported in the literature to date. This anomaly is often discovered incidentally, as in the present case, and may have a myocardial‐protective effect in the presence of coronary artery disease. Conversely, it can also be associated with coronary steal phenomena. However, establishing proof for these mechanisms remains challenging.

## 1. Introduction

Congenital coronary anomalies (CCA) include numerous anatomical peculiarities of the coronary arterial network [1]. An intercoronary communication (ICC), also known as an intercoronary connection or coronary arcade, is a very rare distal CCA (anomaly of termination) characterized by an open arterial communication with unidirectional or bidirectional flow between two main coronary arteries [2]. It consists of evident communications between the right and left coronary arteries through a single, wide (> 1 mm), straight or gently curved vessel. This CCA should not be confused with a collateral circulation that develops secondary to obstructive coronary artery disease (CAD).

## 2. Case Presentation

We report the case of a 41‐year‐old hypertensive female with otherwise an unremarkable medical history. She presented to the emergency department with dyspnea for 3 days and elevated D‐dimer levels (1600 *μ*g/L). Upon admission to the emergency room, the patient was afebrile and normotensive (105/65 mmHg), with an oxygen saturation of 96% on room air. Electrocardiography showed sinus tachycardia at a rate of 110 bpm, with no ST‐T changes. Physical examination revealed an edematous and painful right lower limb with no signs of right‐sided heart failure or pulmonary crackles on auscultation. Pulmonary CT angiography demonstrated a right segmental pulmonary embolism and significantly enlarged mediastinal lymph nodes without pulmonary parenchymal involvement. Doppler ultrasound revealed a deep thrombosis in the right common femoral vein. A transthoracic echocardiogram (TTE) demonstrated a normal right ventricle and pulmonary arterial pressure. However, it revealed apical akinesia and mildly reduced left systolic ventricular function, with an ejection fraction of 45%, and low left ventricular filling pressures. Laboratory tests showed an elevated N‐terminal pro brain natriuretic peptide (NT‐proBNP) level of 4000 pg/mL (normal value < 300) with normal serum creatinine and troponin levels. Coronary angiography was planned to exclude an ischemic etiology for the abnormal TTE findings. Coronary angiography showed no evidence of obstructive CAD but identified an abnormal continuity between the distal right coronary artery (RCA) and the distal left circumflex (LCx) artery at the level of the crux of the heart (Figure 1). As a result, the posterior descending artery was supplied by both coronary systems. The coronary circulation was codominant. Each coronary injection demonstrated a rapid retrograde filling of the contralateral artery, with nearly complete filling observed in the left artery and partial filling in the right artery (Videos S1 and S2). This ICC did not exhibit characteristics typically associated with acquired collateral circulation. Left ventriculography showed a large apical akinesia. Cardiac magnetic resonance imaging (MRI) revealed mild left ventricular dilation, apical akinesia without thrombus, and a left ventricular ejection fraction of 47%. There was no evidence of myocardial oedema or active inflammation. Additionally, multifocal areas of subepicardial late gadolinium enhancement (LGE) were identified in anterior, apical, inferior and lateral segments, consistent with a nonischemic LGE pattern. These MRI findings, combined with the mediastinal lymphadenopathy identified on chest CT, may be suggestive of cardiac sarcoidosis. However, definitive diagnosis requires histopathological confirmation. Anticoagulation with a direct oral anticoagulant was initiated in conjunction with the standard treatment (angiotensin‐converting enzyme inhibitor, beta‐blocker, aldosterone antagonist, and sodium‐glucose cotransporter‐2 inhibitor) for cardiomyopathy with reduced left ventricular function. During hospitalization, the patient′s clinical condition progressively improved, with resolution of dyspnea and reduction of the right lower‐limb edema. NT‐proBNP levels decreased, whereas renal function and serum electrolytes remained stable following initiation of heart failure therapy. The patient was subsequently transferred to the internal medicine department for further evaluation of her venous thromboembolic disease and the cardiac abnormalities identified during the initial assessment. Our specialized multidisciplinary team in CCA concluded that the patient had congenital ICC, which was incidentally discovered and was not associated with an increased risk of myocardial ischemia. The absence of obstructive CAD, the presence of bidirectional coronary flow, and the LGE pattern, which was inconsistent with an ischemic coronary territory, all supported the conclusion that the ICC was unrelated to the patient′s cardiac abnormalities.

**Figure 1:**
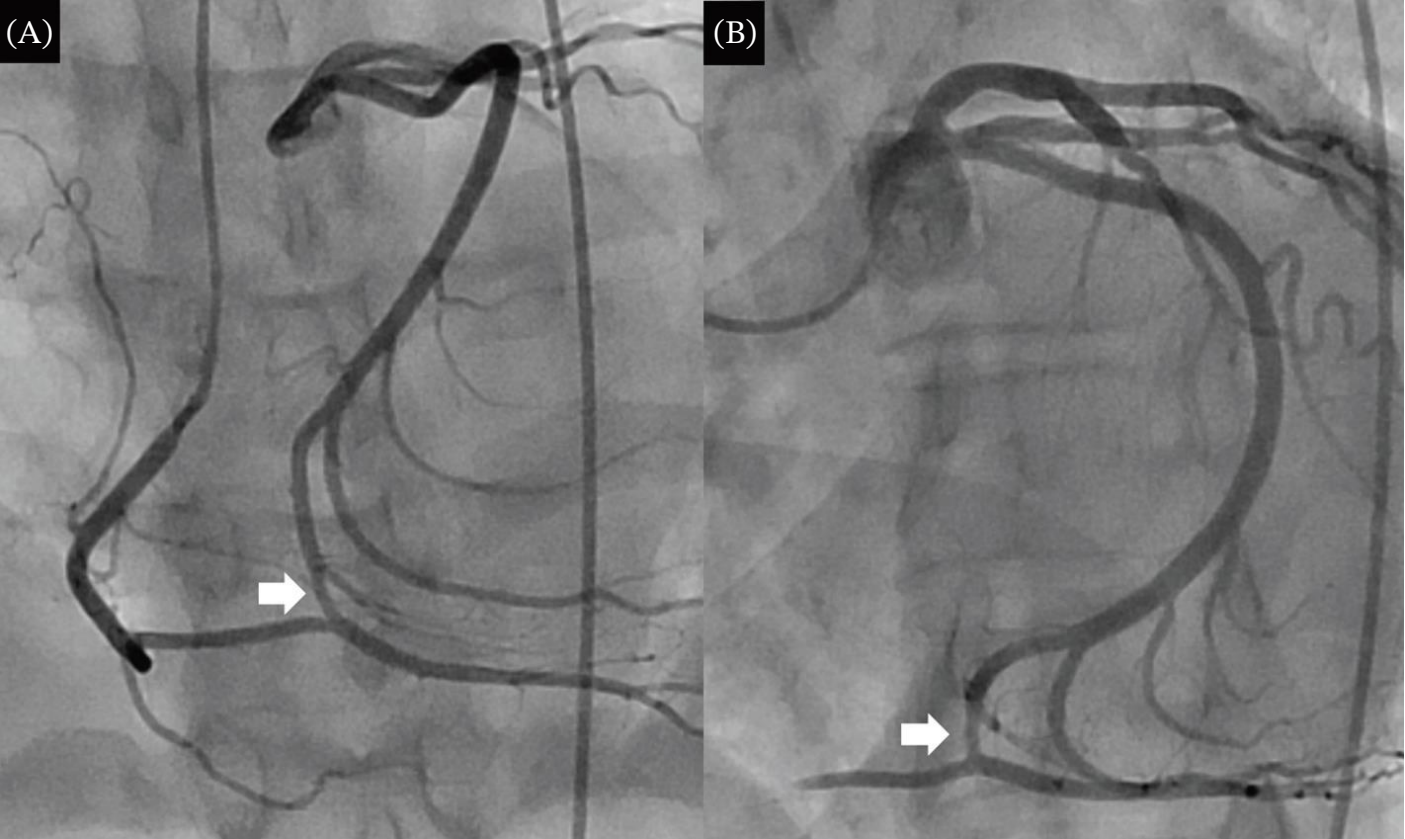
Coronary angiography. (A) Selective right coronary injection with angiographic image (anteroposterior view) showing a short intercoronary communication (white arrow) between the distal right coronary artery and the distal left circumflex artery. Note the retrograde filling of the left circumflex artery and the proximal left coronary network. (B) Selective left coronary injection with angiographic image (left anterior oblique view) showing the intercoronary communication (white arrow), along with a partial retrograde filling of the right coronary artery.

## 3. Discussion

ICC was first documented in vivo by angiography in 1972 by Cheng [3]. A normal coronary network, as seen on angiography, is characterized by anterograde coronary filling and a distinct separation between the two coronary systems. In contrast, an ICC enables both anterograde and retrograde filling of the two coronary arteries, each of which arises from a normal ostium and follows its usual anatomical course, unlike a single coronary artery, which has a single ostium and exhibits a unique retrograde filling of the contralateral artery [4]. Histologically, ICC appears as a normal coronary vessel with similar characteristics, including the three wall layers. A diameter > 1 mm, a course within the atrioventricular or interventricular grooves, and a short and straight trajectory are the key anatomical criteria distinguishing an ICC from acquired collateral circulation that develops between a patent and an obstructed vessel. A defective embryonic development may account for the persistence of a remnant of fetal circulation. True unidirectional flow is rare, while bidirectional flow may exhibit variable retrograde filling, ranging from a few millimeters to the entire vessel. Two types of ICC have been described in the literature; the more common occurs in the atrioventricular groove between the RCA and the LCx artery, whereas the other located in the interventricular groove in the vicinity of the crux cordis between the left anterior descending artery and the posterior descending artery has only been observed postmortem [5]. The exact prevalence of this congenital anomaly is unknown. In the largest study conducted by Yamanaka et al. [6], involving 126,595 patients undergoing coronary angiography, only three patients (0.002%) with this type of anomaly were identified. A comprehensive search of MEDLINE (PubMed) was performed using the following keywords: anomalous coronary artery, congenital coronary artery, coronary arcade, intercoronary communication, and intercoronary connection. All angiographically (selective angiography or CT angiography) confirmed case reports of ICC were included. Reports were excluded if angiographic images were unavailable. Between 1972 and 2025, a total of 28 eligible cases were identified [2, 3, 7–27, 2, 28–30] and described in Table 1. An ICC between the RCA and the LCx artery was observed in all cases. CAD or coronary spasm affecting either the donor or recipient artery was identified in six cases. In reported cases, the causal link between ICC and the initial clinical presentation, such as in our patient, remains uncertain or unestablished.

**Table 1:** Table summarizing all cases of intercoronary communication documented by selective coronary angiography or coronary computed tomography angiography.

No.	Author	Year	Age (years)	Connected arteries	CAD or spasm	Initial presentation	Link with presentation
1	Cheng [3]	1972	48	RCA‐LCx	No	Exertional dyspnea	No
2	Weiner [7]	1979	56	RCA‐LCx	No	Atypical chest pain	No
3	Hines [8]	1981	43	RCA‐LCx	No	Atypical chest pain	No
4	Hines [8]	1981	67	RCA‐LCx	CAD (LAD‐LCx‐RCA)	Angina and heart failure	No
5	Esente [9]	1983	56	RCA‐LCx	No	Chest pain	No
6	Burri [10]	1986	54	RCA‐LCx	No	Atypical chest pain	No
7	Celano [11]	1987	36	RCA‐LCx	No	Atypical chest pain	No
8	Carangal [12]	2000	55	RCA‐LCx	CAD (LAD)	Exertional chest pain	No
9	Atak [13]	2002	49	RCA‐LCx	No	Atypical chest pain	No
10	Arat‐Özkan [14]	2004	18	RCA‐LCx	No	Atypical chest pain	No
11	Gur [15]	2006	43	RCA‐LCx	No	Exertional angina with positive treadmill test	Possible
12	Ulus [16]	2011	53	RCA‐LCx	No	Exertional angina with inferior perfusion defect	Possible
13	Şengül [17]	2011	58	RCA‐LCx	No	Exertional chest pain with apical and inferolateral perfusion defects	Possible
14	Shashanka [18]	2012	41	RCA‐LCx	No	Exertional angina with positive treadmill test	Possible
15	Lee [19]	2012	72	RCA‐LCx	CAD (RCA)	Exertional angina with positive treadmill test	No
16	Kim [20]	2013	45	RCA‐LCx	Spasm (LAD)	Anterior STEMI	No
17	Yildiz [21]	2013	58	RCA‐LCx	No	Angina with positive treadmill test	Possible
18	Abreu [22]	2014	65	RCA‐LCx	No	Atypical chest pain with exertional premature ventricular beats	No
19	Oliveira [23]	2016	58	RCA‐LCx	No	Transient ST segment elevation in lateral leads with inferior mirror	No
20	Gholoobi [24]	2016	57	RCA‐LCx	No	Chest pain	No
21	Baskaran [25]	2019	60	RCA‐LCx	Spasm (LAD‐RCA)	Syncope with transient ST segment elevation in inferolateral leads	No
22	Bath [26]	2019	53	RCA‐LCx	CAD (LAD)	Exertional angina with inferior myocardial perfusion defect	Possible
23	Gan [27]	2019	53	RCA‐LCx	No	Inferior NSTEMI with troponin elevation	Possible
24	Gan [27]	2019	76	RCA‐LCx	CAD (LAD‐LCx‐RCA)	Chest pain	No
25	Ku [2]	2022	36	RCA‐LCx	No	Atypical angina	No
26	Nakamura [28]	2022	30	RCA‐LCx	Spasm (RCA)	At rest chest pain	No
27	Adimoulame [29]	2024	56	RCA‐LCx	CAD (obtuse marginal artery)	Lateral NSTEMI with troponin elevation	No
28	Xiong [30]	2025	77	RCA‐LCx	CAD (LCx‐ RCA)	At rest chest pain	No

Abbreviations: CAD, coronary artery disease; LAD, left anterior descending; LCx, left circumflex; NSTEMI, non‐ST‐elevation myocardial infarction; RCA, right coronary artery; STEMI, ST‐elevation myocardial infarction.

## 4. Conclusion

The clinical significance of ICC remains uncertain. This anomaly may function as a natural bypass, offering myocardial protection in cases of significant coronary stenosis or acute coronary occlusion. Conversely, it could contribute to myocardial ischemia through a coronary steal phenomenon, resulting in inadequate perfusion, particularly if the flow appears unidirectional on angiography. However, even when angina is typical, ischemic tests may yield inconclusive results.

**Nomenclature**
CAD: coronary artery disease
CCA: congenital coronary anomalies
CT: computed tomography
ICC: intercoronary communication
LCx: left circumflex
LGE: late gadolinium enhancement
MRI: magnetic resonance imaging
NT‐proBNP: N‐terminal pro brain natriuretic peptide
RCA: right coronary artery
TTE: transthoracic echocardiography

## Author Contributions

Yassine Ettagmouti: design and written—draft. Amine Bejar: written—review. Asmaa Mounssif: written—review. Ayoub Huda: written—review. Nabil Poulos: written—review. Pierre Aubry: conception, written—review and edition.

## Funding

No funding was received for this manuscript.

## Ethics Statement

Review board approval was not required for this single‐patient case report in accordance with institutional policy.

## Consent

Informed consent for the publication has been obtained from the patient. All images were deidentified prior to submission.

## Conflicts of Interest

The authors declare no conflicts of interest.

## Supporting Information

Additional supporting information can be found online in the Supporting Information section.

## Supporting information


**Supporting Information 1** Video S1: Selective right coronary injection showing a retrograde filling of the left circumflex artery and the left main via a short intercoronary communication between the distal right coronary artery and the distal left circumflex artery.

**Supporting Information 2** Video S2: Selective left coronary injection showing the intercoronary communication, along with a partial retrograde filling of the right coronary artery.

**Supporting Information 3** Data S3: CARE Checklist of information to include when writing a case report.

## Data Availability

The data that support the findings of this study are available on request from the corresponding author. The data are not publicly available due to privacy or ethical restrictions.
